# Sex and Gender Considerations in Episodic Migraine

**DOI:** 10.1007/s11916-022-01052-8

**Published:** 2022-06-09

**Authors:** Sarah R. Ahmad, Nicole Rosendale

**Affiliations:** 1 Headache Division, University of California, San Francisco, CA USA; 2 Neurohospitalist Division, University of California, San Francisco, CA USA

**Keywords:** Gender, Sex, Episodic migraine, Gender minority, Sex hormones, CGRP

## Abstract

**Purpose of Review:**

We seek to update readers on recent advances in our understanding of sex and gender in episodic migraine with a two part series. In part 1, we examine migraine epidemiology in the context of sex and gender, differences in symptomatology, and the influence of sex hormones on migraine pathophysiology (including CGRP). In part 2, we focus on practical clinical considerations for sex and gender in episodic migraine by addressing menstrual migraine and the controversial topic of hormone-containing therapies. We make note of data applicable to gender minority populations, when available, and summarize knowledge on gender affirming hormone therapy and migraine management in transgender individuals. Finally, we briefly address health disparities, socioeconomic considerations, and research bias.

**Recent Findings:**

Migraine is known to be more prevalent, frequent, and disabling in women. There are also differences in migraine co-morbidities and symptomatology. For instance, women are likely to experience more migraine associated symptoms such as nausea, photophobia, and phonophobia. Migraine pathophysiology is influenced by sex hormones, e.g., estrogen withdrawal as a known trigger for migraine. Other hormones such as progesterone and testosterone are less well studied. Relationships between CGRP (the target of new acute and preventive migraine treatments) and sex hormones have been established with both animal and human model studies. The natural course of migraine throughout the lifetime suggests a contribution from hormonal changes, from puberty to pregnancy to menopause/post-menopause. Treatment of menstrual migraine and the use of hormone-containing therapies remains controversial. Re-evaluation of the data reveals that stroke risk is an estrogen dose- and aura frequency-dependent phenomenon. There are limited data on episodic migraine in gender minorities. Gender affirming hormone therapy may be associated with a change in migraine and unique risks (including ischemic stroke with high dose estrogen).

**Summary:**

There are key differences in migraine epidemiology and symptomatology, thought to be driven at least in part by sex hormones which influence migraine pathophysiology and the natural course of migraine throughout the lifetime. More effective and specific treatments for menstrual migraine are needed. A careful examination of the data on estrogen and stroke risk suggests a nuanced approach to the issue of estrogen-containing contraception and hormone replacement therapy is warranted. Our understanding of sex and gender is evolving, with limited but growing research on the relationship between gender affirming therapy and migraine, and treatment considerations for transgender people with migraine.

## Part 1 – Epidemiology and Pathophysiology of Sex and Gender in Episodic Migraine

### Introduction to Sex, Gender, and Episodic Migraine

Social and scientific understanding of sex and gender has evolved over the decades. The distinction between sex and gender was first noted in the 1950s by psychologist John Money and colleagues, who asserted that sex reflected physical characteristics, while gender was defined as one’s behavior and psychological characteristics [1–3]. While definitions may vary, for the purposes of understanding sex and gender in episodic migraine, we will use the currently accepted definitions from the American Psychological Association (APA) guidelines on sexual orientation and gender diversity [4]:

- Sex: *“Refers to a person’s biological status and is typically categorized as male, female, or intersex.”* Indicators of biological sex include sex chromosomes, gonads, internal reproductive organs, and external genitalia.
- Gender: *“Refers to the attitudes, feelings, and behaviors that a given culture associates with a person’s biological sex.”*

Despite the distinction of sex as a biological construct and gender as a social construct, medical research often continues to conflate the two. This lack of clarity can affect our understanding of migraine epidemiology and pathophysiology and can have significant ramifications for understanding migraine in gender diverse populations. Most studies collect either sex or gender in a binary formulation (e.g., female/male or woman/man) and do not distinguish between sex assigned at birth and gender identity. Sex assigned at birth (SAAB) is a biological construct and is assigned based on physical appearance at birth. Gender identity, on the other hand, is independent from (but often concordant with) SAAB and is someone’s internal sense of masculinity, femininity, both, or neither [4]. Gender identity is also independent from gender expression, which refers to someone’s external presentation of gender through mannerisms, clothing, voice, etc. [4] Every individual holds each of these independent facets of identity. Each of these aspects of identity is also independent from sexual orientation, which refers to someone’s romantic, sexual, or emotional attraction to others [4]. Few studies in migraine distinguish between SAAB and gender identity, nor do they include measures of gender expression or sexual orientation. This practice limits our ability to distinguish if differences described in migraine between women and men are due to chromosomal differences, hormonal differences, differential exposure to psychosocial stressors related to gender identity or expression, or a combination thereof.

This practice also limits our understanding of migraine in gender minority (GM) individuals. GM is an umbrella term that describes individuals whose gender identity/expression differs from dominant societal expectations based on SAAB. This term includes, but is not limited to, those who identify as transgender, gender fluid, and gender non-binary [5]. Gender identity distinct from SAAB is not routinely collected in population health surveys, meaning that our understanding of migraine in the GM community is limited to small observational studies with high potential for bias.

In this two-part review, we will be specific about use of sex versus gender when possible; however, a majority of studies cited use these terms interchangeably, and their methodology may not clearly indicate whether gender or sex was studied. In consideration of space limitations, the terms of woman/female and man/male will refer to cisgender (non-transgender) women and men with GM populations specified when applicable. We will review epidemiology, co-morbid conditions, and migraine symptomatology in relation to sex and gender. We will also review the influence of sex hormones on migraine pathophysiology and the use of hormone-containing therapies for patients with migraine. Throughout, we will highlight data relevant to GM as well as briefly review gender affirming hormone therapy and issues unique to migraine in transgender people.

### Epidemiology

In the US and world populations, migraine prevalence is two to three times higher in women [6]. According to the most recent iteration of the Global Burden of Diseases Study (2019), migraine is the most disabling condition affecting young women [7]. In the USA, migraine epidemiology data are drawn primarily from the two largest longitudinal epidemiology studies of episodic and chronic migraine: American Migraine Prevalence and Prevention (AMPP, 2004–2009) and Chronic Migraine Epidemiology and Outcomes (CaMEO, 2012–2013). Among people with migraine, episodic migraine has been reported as far more prevalent than chronic migraine (AMPP 93%, CaMEO 91%). Headache-related disability assessed by the Migraine Disability Assessment (MIDAS) questionnaire was greater in women than men for both episodic and chronic migraine [8]. In the AMPP, sex differences in multiple domains were identified for migraine in general (defined as migraine or probable migraine according to the then current International Classification of Headache Disorders, 2^nd^ edition (ICHD-2) criteria, not stratified by episodic vs chronic). Of note, the CaMEO studies reference gender; however, the methodology suggests participant sex was actually elicited [9]. For the AMPP, gender is the prevailing term used; however, sex is also referenced [10]. In addition to migraine being more common among women, women were more likely to have MIDAS Grade IV headache-related disability, utilize prescription and non-prescription headache medications, and utilize a prescription medication for depression or anxiety. Higher emergency department or urgent care utilization was also observed [11]. Hypothesized reasons for these differences in disability grade may include persistent inequity in household labor; despite advances in education and career attainment, women (including those who work full time) continue to perform the majority of household tasks [12, 13]. Other reasons include the contribution of menstrual migraine, which can be more intense and therefore more disabling [14]. The CaMEO study revealed interesting patterns in men, including differences in diagnosis of migraine (less likely to be diagnosed in men) and co-morbidities (favoring higher rates of co-morbid cardiac disease and stroke, emphysema, and hypertension in men vs allergies, asthma, temporomandibular disorders, anxiety, and Raynaud’s in women). Attack features also differed, with men generally exhibiting a lower frequency of attacks, less allodynia, and fewer aura symptoms. Interestingly, men with episodic migraine were more likely to transition to chronic migraine. As history of head trauma (which is more common in men) was not assessed, its degree of influence on migraine progression is unknown. Gender differences in seeking migraine care or potentially undiscovered prognostic factors in men were cited as possible contributors [15]. Further study in this area is warranted to corroborate these findings. A comparison of these two large studies, CaMEO and AMPP, can be found in Table 1 of Lipton et al. [8].

The Migraine in America Symptoms and Treatment (MAST) study intends to update epidemiologic knowledge of migraine, and began collecting data in 2016 with a prospective, longitudinal, cross-sectional, online survey design with the broad goal of assessing migraine symptoms, diagnosis, management, and co-morbid conditions. In 2018, data on sex differences revealed findings consistent with prior epidemiologic investigations, with higher disability and more frequent headache days in women compared to men. Importantly, the study did not collect inclusive data (elicited as male/men or female/women, sex/gender used interchangeably). Approximately 63% of the sample had a headache frequency consistent with episodic migraine (1–4 monthly headache days (MHD)) and ~ 10% had at least 15, consistent with chronic migraine. Women were over-represented in the chronic migraine and high-frequency episodic migraine (10–14 MHD) groups [16].

Adding to these epidemiologic studies, the ObserVational survey of the Epidemiology, tReatment and Care Of MigrainE (OVERCOME) Study is a prospective online survey study that began enrollment in 2018. Thus far, the Spring 2019 sample of over 20,000 people meeting criteria for migraine demonstrated that optimal acute migraine treatment is associated with lower disability and improved health related quality of life [17]. Sex and gender-specific findings are not yet published, but according to the study methodology, data on gender minorities were not collected.

Our understanding of the epidemiology of migraine in GM populations is significantly limited. A 2021 scoping review of SGM literature in neurology identified only 6 headache articles published from 1960–2020: 4 case reports and 2 cross-sectional studies [18••]. Of these, only the cross-sectional studies examined migraine, and only one included transgender individuals. Using data from The Gender Team Clinic in The Netherlands, Pringsheim and Gooren found that migraine prevalence in transgender women taking gender affirming hormone therapy was similar to that of cisgender women in population estimates, approximately 26% [19]. As this is a single center with only 50 respondents, generalizability is limited. Given limited data sources inclusive of gender identity, we do not yet know the prevalence of migraine in the broader GM community. Future research efforts may help to illuminate these epidemiologic data gaps.

### Migraine Comorbidities

Comorbidity is defined as the association between two conditions in an individual, with the conditions related to one another based on more than chance alone. In migraine, the study of comorbidities may help to illuminate underlying pathophysiologic mechanisms, inform treatment, and identify risk factors for disease progression. According to the most recent epidemiologic data from the MAST study, insomnia, depression, anxiety, and gastric ulcer/GI bleeding are the most common co-morbid conditions. Social and demographic factors modify risk of psychiatric co-morbidities. For instance, those who are married, employed, male sex, and older (≥ 65) have decreased odds of co-morbid anxiety. Similarly, depression is less likely in those with increasing age who are married and employed with higher household income. For relevancy to episodic migraine, higher MHD frequency was associated with higher risk of co-morbidities, both psychiatric and non-psychiatric [20]. There are insufficient data to comment on comorbidities in GM people with migraine.

### Migraine Symptomatology

According to the current ICHD-3, migraine is defined by key characteristics including location (unilateral), quality (pulsating or throbbing), intensity (moderate or severe), duration (greater than 4 h), and associated symptoms (nausea and/or vomiting, photophobia and phonophobia). Anecdotally, migraine phenotypes vary widely from person to person, with some more significantly impacted by associated nausea or photophobia than by pain itself, or vice versa. One of the earliest studies specifically examining the impact of gender on migraine features demonstrated that nausea, photophobia, and phonophobia were more prevalent in women; additionally, migraine attack duration and intensity were higher. Age-dependent variations in symptomatology were noted for women, but not for men [21]. Of note, in this study, the term gender is used; however, it is not clear whether the authors assessed sex or gender per APA definitions. Data from the AMPP demonstrate similar sex differences. The majority of respondents experienced episodic migraine (1–4 MHDs). Average pain intensity was similar between sexes; however, women were more likely to experience most migraine criteria symptoms (nausea, vomiting, photophobia, phonophobia, visual aura). In terms of function, women were more likely to report requiring bed rest and experience a longer duration of impairment in function after a migraine attack compared to men, who more often reported being able to continue to function, and with shorter duration of post-migraine impairment [22].

### Pathophysiologic Considerations: Hormones

Despite clear epidemiologic differences in migraine by sex, underlying mechanisms for this pattern remain poorly understood; however, sex hormones are thought to contribute. Throughout the recent decades, knowledge of the underlying mechanisms of migraine has evolved, revealing a complex set of processes including activation of functional neural networks, vascular changes, hyperexcitability, and neurotransmitter and neuropeptide signaling. Sex hormones play a role in aspects of migraine pathogenesis, which is reviewed here.

#### Migraine Pathogenesis

Migraine attacks begin with a premonitory phase of hypothalamic, brainstem, and cortical activation correlating with symptoms of mood changes, fatigue, food cravings, yawning, neck pain, or sensitivity to stimuli (light, sound). Cortical spreading depolarization (a wave of neuronal depolarization), thought to underlie aura, consists of reversible neurologic symptoms (most commonly visual) that typically occur prior to onset of headache pain. Aura may also occur at the onset of the headache phase or persist throughout. Headache pain is generated by trigeminovascular activation. The trigeminovascular system is a functional pathway which transmits nociception from the meninges and cerebral arteries to the brainstem trigeminocervical complex (TCC) (comprising the trigeminal nucleus caudalis and upper cervical dorsal horn), then to the hypothalamus, thalamus, and cortical regions. Pain referral patterns in the head and neck are complex given convergence of sensory and nociceptive inputs from the trigeminal ganglion and other trigeminal and cervical structures, which produces pain perception in a variety of regions of the face, head, and neck. Cortical processing of pain inputs likely generates the migraine-associated symptoms including sensitivity to stimuli and cognitive symptoms. When the trigeminovascular system is activated, dural nociceptive afferents are stimulated, releasing the potent vasodilator, calcitonin gene-related peptide (CGRP), along with substance P and pituitary adenylate cyclase-activating polypeptide-38, into the perivascular space. This generates neurogenic vasodilation. CGRP also acts at other locations, including the trigeminal ganglion and TCC. Peripheral sensitization of trigeminovascular neurons is characterized by a reduction in the threshold of responses and an increase in the magnitude of responses to dural stimuli. This process may underlie migraine pain characteristics including throbbing quality and exacerbation with movement. CGRP and other factors may play a role. Repetitive activation of the trigeminovascular pathway is thought to generate central sensitization, which clinically manifests as cutaneous allodynia (cephalic and extracephalic) and may contribute to chronification. Details of migraine pathophysiology are complex and remain in flux as new insights from basic science and functional imaging research are gathered; these concepts are further reviewed in detail in dedicated reviews (see [23, 24]).

#### Hormones and Migraine

The first investigation of the relationship between hormonal changes and migraine was published by Somerville in 1972, who proposed that estrogen withdrawal may trigger migraine [25]. Subsequent research by MacGregor and others have similarly shown that migraine attacks are associated with a decline in estrogen in the late luteal (pre-menstrual) phase of the menstrual cycle [26, 27]. A recent investigation of sex hormone levels found that the rate of estrogen decline in the luteal phase was more precipitous in persons with migraine compared to controls; however, this decline is not always associated with a migraine attack, suggesting it is a susceptibility factor that may facilitate migraine if triggered by other factors (change in sleep, stress, or other individual triggers) [28]. A decline in exogenously administered estrogen was only associated with triggering migraine in those women with pre-menopausal history of migraine [29], suggesting an underlying vulnerability to hormonal fluctuations in those with migraine. An interesting recent exploration of levels of sex hormones in non-obese men with migraine demonstrated higher levels of estradiol (and clinical findings of androgen deficiency) [30]. In animal models, estrogen may increase responses in the trigeminovascular system [31] and increase susceptibility to cortical spreading depolarization [32]. Clinically, pregnancy and use of hormone replacement therapy or hormonal contraception are high estrogen states associated with an increase in migraine with aura [33].

Other hormones including progesterone and testosterone may also play a role in migraine, although are less well studied than estrogen. Circulating hormones (including estrogen, progesterone, and testosterone) are able to penetrate the blood brain barrier as lipophilic molecules. There, they function as precursors for neurosteroids, which are synthesized in the central nervous system. For progesterone, this mechanism is important to acknowledge, because allopregnanolone (a derivative of progesterone and pregnenolone) is the centrally acting progesterone in the CNS which enhances GABA function (as a GABA receptor modulator), thereby inhibiting neuronal excitability [34, 35]. In animal models of epilepsy, withdrawal of progesterone reduces the seizure threshold by this mechanism, which is thought to underlie catamenial epilepsy [36]. As in epilepsy, cortical excitability also contributes to migraine. Overall, progesterone is thought to have a neuroprotective effect by reducing nociception in the trigeminovascular system. A recently published cross-sectional pilot study demonstrated that women with migraine had lower serum allopregnanolone levels (despite similar serum levels of progesterone between those with migraine and controls) with an inverse relationship to the years and frequency of migraine [37]. This finding may be related to allopregnanolone’s neuroprotective effects, which may reduce neurogenic inflammation in migraine (that can, in turn, contribute to central sensitization and chronification).

The role of testosterone in migraine is less well understood. A prospective pilot study of subcutaneous testosterone implants in women (both pre- and post-menopausal) with a diagnosis of migraine (episodic vs chronic not specified) demonstrated improvement in headache intensity. Importantly, there are significant limitations to this study, as the patient population had symptoms of androgen deficiency, there was no control group (degree of placebo effect unknown), and the outcome was an assessment of headache intensity (5-point scale) rather than interval assessment of headache frequency (MHDs) or use of a validated instrument to measure change after a therapeutic intervention (HIT-6, MIDAS) [38]. Another small study of danazol (an androgen) demonstrated improvement in “hormonal migraine” (menstrual migraine) [39]. A recent prospective observational pilot study demonstrated that men with chronic migraine had lower testosterone compared to controls [40]. Underlying mechanisms of testosterone in migraine may include suppression of cortical spreading depolarization [41], increase in serotonin [42], stabilization of cerebral blood flow [43], and neuroprotective [44] and anti-inflammatory effects [45]. In the Dutch study of migraine in transgender women taking hormone therapy (antiandrogens and estrogens), there were high rates of migraine with aura, consistent with prior observations of migraine aura developing while using estrogen replacement therapy in cisgender women [46].

#### Hormones and CGRP

With the recent advent of migraine-specific acute and preventive therapies targeting CGRP, relationships between CGRP and sex hormones have been examined in animal models. Broadly, current knowledge suggests that estrogen receptors are expressed in the trigeminovascular system [47] and cyclical changes in estrogen levels can influence CGRP release and receptor signaling. Animal studies have demonstrated that activation of the CGRP system varies with the estrous cycle [48], and estrogens may regulate the excitability and sensitization of the CGRP pathway (reviewed in detail [49••]). Additionally, in rat models, estradiol was shown to increase neurogenic vasodilation [31, 50] and contribute to cortical spreading depolarization by way of the estrogen receptor [51]. In humans, an experimental model of CRGRP release from sensory neurons in the dermis initiated by capsaicin demonstrated that in healthy women, there was increased reactivity of this response when estrogen levels were low. However, in women with migraine, while responses were elevated compared to participants without migraine, there was no variation with the menstrual cycle [52]. This suggests a heightened CGRP response in individuals with migraine and an effect of estrogen on CGRP, consistent with prior research demonstrating a decline in estrogen is associated with susceptibility to migraine. In addition to menstrual fluctuation of estrogen, there is a progressive increase in prostaglandin levels from the follicular to luteal phase and into menstruation [53], which promotes the release of substance P, neurokinins, and CGRP in neurogenic inflammation (updated review in [54]). Pregnancy [55, 56] and menopause [57], phases characterized by changes in sex hormones, are associated with changes in circulating CGRP, which reflects the interaction between hormones and CGRP.

#### Natural Course of Migraine and Hormonal Intersections

Differences in onset and prevalence of migraine by gender and life phase suggest an underlying contribution from hormone changes. For example, in children prior to puberty, the one-year prevalence is similar for boys and girls for age 7–9 (range 2–5%) and age 10–12 (4–5%). At puberty, this pattern diverges. Prevalence increases for boys and girls, but is more pronounced in girls (6% vs 4%). Migraine continues to remain far more prevalent throughout the lifetime for women [6]. Peaks in migraine prevalence occur at approximately age 35 and age 50 for both sexes, with a tendency to decline with increased age, although for men the natural course is relatively more stable throughout the lifetime [58••]. Other patterns that demonstrate evidence of a hormonal contribution to migraine include a high prevalence of women with menstrually related migraine (at least 20% of women with migraine) [59] and a change in migraine (usually improvement) with pregnancy [60] and menopause [61, 62]. Interestingly, women with menstrual migraine may be more likely to experience improvement in migraine during pregnancy [60] and a higher frequency and intensity of migraine in the perimenopausal period [61, 63]. In perimenopause, fluctuations in estrogen and progesterone, specifically more frequent and longer periods of estrogen withdrawal, are thought to contribute [26, 64]. Migraine frequency tends to decline in menopause [58••].

### Conclusion

Epidemiologically, migraine is known to be more prevalent and disabling in women, and some data suggest an increased prevalence of migraine in transfeminine individuals. Additional epidemiologic investigation is required to better understand migraine in GM individuals. Migraine co-morbidities and symptomatology also appear to vary by sex. These differences are thought to be accounted for by underlying sex hormones. Estrogen is the most well-studied sex hormone, and in animal and human models, influences aspects of migraine pathogenesis from cortical spreading depolarization to trigeminovascular activation and CGRP signaling. The natural course of migraine throughout the lifetime also reflects hormonal influences, with migraine prevalence diverging at puberty for children, and variations in migraine frequency and intensity with pregnancy and menopause.

## Part 2 – Practical Considerations for Sex and Gender Issues in Episodic Migraine

### Introduction

With the knowledge of the epidemiology and pathophysiologic basis for sex differences in migraine, mediated in large part by sex hormones, we can now address the clinical implications of this knowledge. We first address menstrual migraine, a diagnosis which, to date, remains in the appendix of the ICHD-3. Menstrual migraine is common, and unfortunately more severe and disabling, whether purely menstrual migraine (only occurring in association with menstruation) or menstrually related (both perimenstrual attacks and attacks at other times). Despite this, there are no specific approved treatments for menstrual migraine. Oral contraceptives and hormone replacement therapies containing estrogen have traditionally been recommended against for people with migraine with aura; however, a re-examination of the data underlying this practice suggests that a more nuanced approach may be more appropriate. Finally, data on gender affirming hormone therapy and treatment of migraine in transgender individuals are limited. We highlight the known practical considerations of understanding gender affirming hormone therapy in the context of migraine, and unique aspects of treatment of migraine in transgender people. Please refer to Part 1 for additional background information and details on terminology.

### Menstrual Migraine

Diagnostic criteria for menstrual migraine remain in the appendix of the ICHD-3, pending further study and validation. Pure menstrual migraine is defined by perimenstrual attacks only (occurring prior to or during menstruation), while menstrually related migraine consists of both perimenstrual attacks and attacks at other points of the cycle. For episodic migraine, given the relatively lower frequency of attacks, these definitions are sufficient. However, in chronic migraine, it is quite likely that a person will have a migraine attack coincidentally in the peri-menstrual period, and could meet criteria for menstrually related migraine. In research, study designs may differ in their definitions of menstrual migraine, making uniform comparisons difficult and estimates of prevalence variable. Study population also influences prevalence estimates. In the headache clinic population, menstrual migraine (without aura) has a prevalence of 22–70% [65–67], higher than estimates from the general population, which range from 18–25% of individuals with migraine [62, 63, 68, 69] Studies citing a higher prevalence typically have less restrictive definitions of menstrual migraine [59]. Another complicating factor is the effect of oral contraceptive use, as headache is common during the placebo/hormone-free week, and may be different from headache occurring in the context of non-hormonally influenced menstrual cycles [70].

A number of studies have shown that perimenstrual migraine attacks are more intense, prolonged, and debilitating, as well as less responsive to treatment and more likely to be accompanied by associated symptoms such as sensitivity to stimuli and nausea [64, 71–77]. However, no treatments are specifically approved for treatment of perimenstrual migraine. For individuals with episodic migraine, the identification of effective acute treatments takes priority. The decision to start prevention for those with episodic migraine and menstrual migraine should take into account the frequency, intensity, and overall burden of disease. In addition to acute or preventive therapies, short-term perimenstrual prevention should be considered. In practice, the off-label use of frovatriptan for prevention of perimenstrual attacks (started a few days prior to onset of expected menstruation and continued during menstruation) is popular and supported by some evidence [78, 79]. Other triptans used for short-term prevention include naratriptan and zolmitriptan [78]. Use of hormonal contraceptives for menstrual migraine is reviewed below.

### Oral Contraceptives and Hormone Replacement Therapy

Despite the pathophysiologic link between hormones and migraine, clear evidence regarding the utility of hormonal treatments in migraine, including oral contraceptives (OCPs) and hormone replacement therapy (HRT) in menopausal women, is limited. If standard acute and preventive therapies remain ineffective in treating menstrual migraine, there is some evidence to support the use of continuous, low dose estrogen-containing contraception, which minimizes fluctuations in estrogen [80], or regimens that limit the decline in estrogen (to < 10 ug) which triggers migraine [81]. Use of estrogen-containing contraception has been controversial due to concerns for ischemic stroke risk, and consequently, individuals with migraine with aura were excluded from these studies of continuous regimen oral contraceptives for menstrual migraine. Migraine with aura carries a twofold increased risk of ischemic stroke even when controlling for other stroke risk factors [82–87]. Traditionally, the prevailing consensus has been to avoid oral estrogen-containing contraception (which may further compound stroke risk) in migraine with aura. If used for the sole purpose of contraception, alternatives (progestin-only, intrauterine devices) are preferred. However, some have challenged this assertion based on a renewed evaluation of the data. The first report of stroke risk with combined OCPs was published in 1975 [88], at a time when estrogen doses were significantly higher (for example, 100–150 ug mestranol, the popular dose for oral contraception in the 1960s-1970s). More recent investigations revealed that stroke risk was dose-dependent, and that low dose formulations [89] were not associated with increased risk [90]. Today, most oral contraceptives use 10–35 ug ethinyl estradiol (only 1% contain 50 ug). Additionally, a large-scale study [91] and pooled analysis of multiple US studies [92] demonstrated no increased risk of stroke with low dose OCPs. In addition to estrogen dose, aura frequency is a factor in risk assessment. The risk of ischemic stroke is directly correlated with frequency of aura [93, 94]. Updated evaluations of the research on combined OCPs and stroke risk are reviewed in detail in two recent reviews by Calhoun and Batur, and Voedisch and Hindiyeh [95••, 96]. In summary, while estrogen-containing OCPs should be avoided when possible in individuals with migraine with aura, aura frequency and estrogen dose should be considered for a more tailored approach. Non-oral hormones may be an alternative option. A clinic-based retrospective study of patients with migraine with aura and intractable menstrually related migraine showed that continuous use of vaginal ring low dose (15 ug/24 h) ethinyl estradiol reduced aura frequency and improved menstrual migraine in > 90% of participants [97].

In menopause, the few studies of HRT and migraine have produced inconsistent results. Most studies assessing stroke risk in migraine with aura comprised younger participants; therefore, there are insufficient data for older women. A single study examining risk of ischemic stroke in women with migraine using HRT showed no significant association; however, details of HRT (type, dose, route) were not available [87]. Low-dose transdermal estrogen is not thought to be associated with any significant increase in stroke [98, 99] and may minimize the estrogen fluctuations associated with triggering migraine while improving the associated vasomotor symptoms of menopause [100••]. In contrast, high doses of oral estrogen are associated with triggering new migraine with aura or worsening existing migraine with aura [101]. If migraine worsens or if new migraine with aura develops, HRT should be discontinued. This topic is reviewed in more detail by MacGregor in a 2018 review [100••].

### Gender Affirming Hormone Therapy and Migraine

Data examining the effect of gender affirming hormone therapy (GAHT) on migraine prevalence or disability are limited. It is important to note that not all GM individuals use GAHT: the decision of which, if any, hormones to use is individual and dependent on the goals for gender affirmation [102]. A single study from Italy examined the influence of GAHT on pain in 47 transgender women and 26 transgender men, in which headache was included as a pain outcome [103]. They found that in 14 transgender women who endorsed ongoing pain, 3 had onset of headache after initiation of GAHT, while 2 experienced headache prior to the initiation of GAHT, although these respondents also reported worsening of headache intensity after initiation. In this sample of transgender men, 16 reported ongoing pain, 13 of whom endorsed headache. The majority (10) had headache prior to initiation of GAHT with varied responses after initiation of testosterone: headache improved in 6, remained unchanged in 3, and worsened in 1 respondent. This study did not define headache type, although does describe associated photophobia and phonophobia with headache in the transgender women and a family history of headache in the transmen respondents with headache. It also remains unknown how or if gender affirming surgeries influence migraine prevalence and severity.

Given the limited data, management of migraine in transgender people using GAHT remains largely based on consensus. In transfeminine people, it is important to maintain steady levels of estrogen, often best accomplished with non-oral formulations of estrogen, and to monitor estrogen levels to ensure they remain within physiologic ranges [104••]. In transmasculine people, estrogen fluctuations from residual ovarian activity may be sufficient to provoke migraine even in the absence of menstruation. The addition of intramuscular medroxyprogesterone acetate may be beneficial in this instance [104••]. It is also important to be mindful of the potential interactions between GAHT and migraine preventive therapy, namely antiepileptic therapies. Estrogens, progestins, and testosterone are metabolized through the CYP 3A4 pathway, which may be affected by antiepileptic therapies like topiramate and valproic acid [105]. Available data describing this potential interaction are exclusively in cisgender populations, however, so the extent to which this influences clinical care in transgender people is uncertain. Gender affirming therapy is medically necessary and life-saving. If there is concern for a potential interaction, every effort should be made to adjust migraine therapy or alter the formulation/type of GAHT in conjunction with the patient and their clinician prescribing gender affirming care in an effort to best preserve it.

### Management of Migraine in Transgender Individuals

In addition to the standard diagnostic studies and treatments relevant to migraine, there are aspects of care unique to transgender individuals. Providers should understand risks associated with GAHT. For masculinizing hormones (testosterone), there is a potential risk of secondary polycythemia which can in turn present with headache and increased risk of migraine aura [106, 107]. For feminizing hormones, higher doses of oral estrogen may increase risk for venous thrombosis, stroke, and cardiovascular disease. In addition, cyproterone acetate in high doses is associated with prolactinoma and meningioma [108, 109]. Given these potential risks, it is important to remain vigilant for secondary causes of headache. Currently, there are insufficient data on risk of ischemic stroke in transgender women with migraine using estrogen therapy, and risk assessment relies on data from the cisgender women population.

Providers may also observe an increase in migraine and migraine aura in individuals using feminizing hormones given the pathophysiologic impacts of estrogen on the trigeminovascular system and cortical spreading depolarization [110]. Although the influence of testosterone on migraine has not been studied extensively, there is some evidence for decreased risk of migraine in transgender men [103]. Underlying mechanisms from pre-clinical studies may include testosterone’s inhibition of cortical spreading depolarization [111] as well as anti-nociceptive and anti-inflammatory properties of testosterone [112, 113].

Given the increased risk of ischemic stroke in migraine with aura, for transgender individuals using estrogen-containing compounds, providers should advise reduction of modifiable risk factors for stroke such as avoiding tobacco use (known to increase stroke risk [114]) and management of other vascular risk factors (diabetes, hypertension, and hyperlipidemia).

### Health Disparities, Socioeconomic Considerations, and Research Bias

As in other neurologic conditions, structural and social determinants of health affect migraine incidence, prevalence, and outcomes. While a thorough exploration of disparities in migraine is outside the scope of this review, there are particular connections of other personal and social identities with sex and gender that may affect migraine prevalence and severity. This requires an understanding of the concept of intersectionality. Intersectionality refers to the interconnected and overlapping systems of discrimination and disadvantage experienced due to holding multiple minoritized social identities [115]. To our knowledge, there are no intersectional analyses of episodic migraine [116]. Prior studies have found disparate rates of self-reported migraine based on race/ethnicity [117], socioeconomic status [116], and sexual orientation [118]. In addition, factors including income, health insurance status, and gender influence access to adequate headache management [119]. An editorial published in 2006 suggested that pharmaceutical marketing targeted toward women may exacerbate gender bias and create barriers for headache treatment, as men are less likely to seek care for headache and are less likely to be diagnosed with migraine [120]. Further research is needed to expand these analyses and understand the influence of additional and concurrent social determinants of health.

Despite the higher prevalence of migraine in women, animal models of migraine are primarily male, previously justified by limiting the influence of fluctuating sex hormones on outcomes [121]. In contrast, human clinical studies typically have an overrepresentation of women, limiting generalizability to the still sizable proportion of men with migraine. National Institutes of Health funding for the study of sex as a variable in both animal and human studies has increased [122], opening new avenues for future investigation.

### Conclusion

Menstrual migraine lacks a clear definition, and there are no specific treatments for this type of more severe and disabling migraine. Estrogen-containing contraceptives and HRT may increase the risk of ischemic stroke; however, lower dose formulations and transdermal routes of administration in patients with infrequent aura and low vascular risk factor profile may be appropriate. Risks and benefits unique to each patient should be considered. There may be a role for use of hormone-containing treatments for management of menstrual migraine. As concepts of sex and gender have evolved, robust data in headache inclusive of gender minorities remain limited. A majority of studies use gender and sex interchangeably, and limit categorization to binary male/female or man/woman. The paucity of inclusive research offers an opportunity for future exploration to enhance understanding and treatment of migraine in gender diverse populations. Data on gender affirming hormone therapy and migraine are limited and management relies on consensus. Providers should take into account unique aspects of migraine management in transgender populations, including use of feminizing and masculinizing hormones. Finally, as with other medical and neurologic conditions, structural and social determinants of health influence migraine, which may be further explored with an intersectional approach, taking into account sex and gender among other identities.
